# Social Isolation and Loneliness among Near-Centenarians and Centenarians: Results from the Fordham Centenarian Study

**DOI:** 10.3390/ijerph19105940

**Published:** 2022-05-13

**Authors:** Daniele Zaccaria, Stefano Cavalli, Barbara Masotti, Carla Gomes Da Rocha, Armin von Gunten, Daniela S. Jopp

**Affiliations:** 1 Centre of Competence on Ageing, Department of Business Economics, Health and Social Care, University of Applied Sciences and Arts of Southern Switzerland (SUPSI), 6928 Manno, Switzerland; stefano.cavalli@supsi.ch (S.C.); barbara.masotti@supsi.ch (B.M.); 2 Service of Old Age Psychiatry, Lausanne University Hospital, 1011 Lausanne, Switzerland; carla.gomesdarocha@hevs.ch (C.G.D.R.); armin.von-gunten@chuv.ch (A.v.G.); 3 School of Health Sciences, HES-SO Valais-Wallis, 3960 Sion, Switzerland; 4 Institute of Biomedical Sciences Abel Salazar, University of Porto, 4050-313 Porto, Portugal; 5 Institute of Psychology, University of Lausanne, 1015 Lausanne, Switzerland; daniela.jopp@unil.ch; 6 Swiss Centre of Expertise in Life Course Research (LIVES), 1015 Lausanne, Switzerland

**Keywords:** social isolation, loneliness, near-centenarians, centenarians, Fordham Centenarian Study, typology

## Abstract

Social isolation and loneliness have been recognized as problems older people face due to their adverse effects on health and mortality, but very few researchers have analyzed their co-occurrence, which might be particularly prevalent and critical among the very old. In this study, we investigated the prevalence of combinations of social isolation and loneliness among near-centenarians and centenarians. We used data collected from 94 individuals aged 95–107 from the Fordham Centenarian Study. We built a four-group typology and explored associations with individual characteristics in various domains (demographic, socioeconomics, social, health, care, and psychological) with multinomial logistic regression models. Considering their combinations, the most prevalent groups were “isolated and lonely” and “neither isolated nor lonely” (29.8% and 28.7%, respectively). The “lonely but not isolated” (20.2%) and “isolated but not lonely” (21.3%) groups were also notably large. The likelihood of belonging to each group varied according to various individual characteristics, such as education, health, and personality. Social isolation and loneliness are distinct phenomena among centenarians. The consideration of their varied combination can help better assess life conditions at very old ages. Taking into account the differences between groups can facilitate the design of tailored interventions to improve the lives of near-centenarians and centenarians.

## 1. Introduction

Social isolation and loneliness represent important issues in old age as older individuals will likely face the loss of loved ones, including spouses, close family members, and friends [1]. Furthermore, age-related health limitations, sensory impairment, and multimorbidity hinder social contacts and are linked to increased loneliness [2]. In addition, residential relocation, such as moving from a family home to an institution, is common at advanced ages and can lead to the deterioration of long-term social relationships [3]. Consequently, studies on old and very old individuals have shown an increased prevalence of social isolation and loneliness [4,5].

Social isolation and loneliness are associated with adverse implications on health and quality of life among older adults [6]. Being socially isolated implies impaired immune function, poor nutritional habits, and increased risk of coronary diseases and stroke [7,8,9]. Loneliness is a predictor of poor mental health, desire to die, and mortality [10,11,12]. Furthermore, the degree to which social isolation and loneliness impact mortality risk is comparable to that of other well-established risk factors, such as smoking or obesity [13].

Social isolation and loneliness are often considered interchangeable, but they identify different constructs [14]. Social isolation is a concept that refers to the objective characteristics of a situation with a measurable lack of relationships and engagement with other people. Hence, individuals with few meaningful social connections can be considered socially isolated [15]. Loneliness is the subjective feeling of isolation accompanied by the perception of a deficiency in the desired number or quality of social relations [16]. The two concepts are weakly correlated among older adults [17].

In the present study, we investigated social isolation and loneliness in combination, considering a sample of near-centenarians and centenarians. In particular, we explored the correlates of the groups resulting from this combination, drawing mainly on those characteristics previously investigated in several studies on oldest-old and centenarians that were focused on the two phenomena separately.

### 1.1. Correlates of Loneliness and Social Isolation among Oldest-Old and Centenarians

Although loneliness and social isolation are often discussed together and compared to one another, they are usually investigated separately. Various individual characteristics have been associated with social isolation and loneliness among the oldest-old. As previous studies have shown [18,19,20], these variables can be grouped into multiple domains: demographic, socioeconomic, social, health, care, and psychological.

Concerning demographic characteristics, findings suggest that women are at greater risk of being socially isolated or lonely, possibly because they are more likely to be widowed and living alone than men [21]. A recent study on the oldest-old living in New Zealand showed that the risk of loneliness was the same for men and women [22]. Some scholars have also investigated the role of ethnicity. For instance, Finlay and Kobayashi [23], in their study on old and oldest-old in the U.S., found that African Americans were less likely to report social isolation and loneliness than Whites, but the least represented ethnic groups were most at risk of loneliness in a Chinese sample [24] and in a New Zealand sample [22].

In previous studies, socioeconomic factors, such as education or income, have been considered mainly as control variables [25]; however, some scholars have analyzed their association with loneliness, but with mixed results. A recent study on a Chinese oldest-old population showed that lower education and poor economic status were associated with higher loneliness [24]. In contrast, findings from the Georgia Centenarian Study did not indicate any significant association between socioeconomic measures and loneliness [26].

Regarding social characteristics, the loss of a partner has received much attention: widowhood, especially early widowhood, was a strong predictor of increased loneliness in the Newcastle 85+ study [27]. Furthermore, an Australian qualitative study showed an association between early widowhood and increased social isolation, particularly for men [21]. Having children can also be relevant: centenarians’ children were their primary source of support in daily life in the Heidelberg Centenarian study sample [28]; the absence of children’s support was associated with loneliness and social isolation in many studies on the oldest-old [29]. In addition, their proximity to their children and the number of visits received played a role in decreasing feelings of loneliness among Chinese oldest-old [30]. Living conditions had a strong association with social isolation and loneliness: living alone and being institutionalized were associated with a greater risk of being isolated or lonely in European and New Zealand contexts [3,22,31], but the association with living in institutional settings was more controversial in another study on Chinese oldest-old [30]. Finally, gathering with family, friends, and acquaintances showed a significant association with a lower level of social isolation and loneliness in a Norwegian study [32], as did satisfaction with the number and frequency of social contacts in a Swedish oldest-old sample [33].

The individual characteristics that have received the most interest in analyses of social isolation or loneliness concern health. The association between poor health and social isolation has been well documented in several studies [29]. Physical health was found to be a good predictor of loneliness in a U.S. and English sample, but not in a group of Swedish oldest-old [34,35], and subjective health was found to be a strong correlate of loneliness in a Chinese oldest-old sample [24]. As a dimension of health, the functional status has its own impact: a study on a Norwegian oldest-old sample demonstrated that being dependent was related to increased social isolation [32], and a more recent study also conducted in Norway showed a significant relationship between poor functional status and loneliness [36]. In addition, social isolation and loneliness have been found to be associated with health symptoms such as pain and fatigue in two U.S. samples [37]. Finally, the perception of one’s own health as a barrier to preferred activities has been associated with various indicators of the psychological well-being of the oldest-old, including an increased risk of loneliness in the Georgia Centenarian Study [38].

Despite informal home care’s undisputed role as a means of maintaining social contacts in old age [39,40], research addressing this topic among very old people is still scarce and is mainly limited to formal home care services. Indeed, some scholars have shown that reliance on formal services reduced loneliness among the oldest Canadians [41,42], but a recent literature review has shown mixed research evidence [43].

Many psychological characteristics have been considered, but the one that received the most attention is depression. Although depression’s relationship with social isolation in oldest age is not completely clear in various studies [29], it is recognized as one of the most significant risk factors for loneliness in Northern Europe and New Zealand [3,22]. In a Chinese study on various psychological factors, the researchers found a negative correlation between life satisfaction and loneliness in the oldest-old [24]. Finally, certain personality traits seem to be the drivers of loneliness; for instance, loneliness has been linked to high neuroticism [44] and low extraversion [25] in two samples of U.S. oldest-old.

### 1.2. The Combination of Social Isolation and Loneliness

Loneliness is more prevalent in the oldest-old than it is in the younger-old [5], and social isolation tends to grow more severe with increasing age [4]. Therefore, studying the combination of these two aspects among the very old, such as near-centenarians and centenarians, can be particularly worthwhile. Many scholars have reported that older adults are at greater risk of exposure to experiences leading to social isolation and loneliness, such as loss of loved ones [27] or institutionalization [3]. Thus, extremely long-lived individuals may be at even greater risk of experiencing social isolation and loneliness.

The idea that social isolation and loneliness should be examined together has long been of interest to several researchers, who have suggested the need to identify a typology useful for investigating the combined characteristics and outcomes of these two phenomena [45]. Nevertheless, empirical research on the combination of social isolation and loneliness in older adults is limited. Recently, some scholars have started to examine social isolation and loneliness in combination in an effort to understand their association with physical and mental health. More specifically, Hsu and colleagues used a survey on older adults in Taipei, Smith and Victor analyzed data from the English Longitudinal Study of Aging, and the study by Menec and colleagues was based on the Canadian Longitudinal Study on Aging [46,47,48]. However, in all these studies, researchers focused on a wide category of older individuals (i.e., 65 years of age or older) without specific attention to the oldest-old or centenarians. In these studies, despite different methodological approaches and measures of isolation and loneliness, the “neither isolated nor lonely” group (47% to 70%) was the largest, and the “isolated and lonely” group always included the smallest number of individuals (3% to 7%) [46,47,48]. The “neither isolated nor lonely” group was generally characterized by a higher level of education and better physical and mental health, and the “isolated and lonely” group reported worse functional status, more depression, less life satisfaction, and lower socioeconomic status in all studies [47]. Older adults who reported “only isolation (but no loneliness)” (27%; [46]) were more likely to have had children but had lower life satisfaction and were less likely to desire more social engagement. Older adults who reported “only loneliness (but no isolation)” (7%; [48]) were more likely to experience psychological distress and low satisfaction with family relationships.

Although there is still little empirical evidence, these three studies have shown how important it is to consider the combination of social isolation and loneliness. Focusing only on social isolation without considering loneliness (or vice versa) can lead to underestimating potential negative outcomes (e.g., a person may have many contacts but still feel lonely). Analyzing social isolation and loneliness in tandem means acknowledging that specific conditions may be the result of a personal choice (e.g., not wanting more contacts) or an ability to adapt one’s expectations to the reality of a situation (e.g., being happy with very few contacts; [49]). Socially isolated but not lonely individuals may prefer to have only a handful of social relationships, possibly due to a more reserved personality, or they may be able to optimize the relationships they have and adapt their expectations so that isolation does not necessarily result in loneliness [50]. For those who are “lonely in the crowd” [51], being embedded in large social networks may not prevent loneliness. Having many relationships does not automatically mean being satisfied with them [52]: the right people may be missing because they have passed away or live far away [53].

### 1.3. The Present Study

We investigated how many near-centenarians and centenarians experience either social isolation or loneliness, focusing our attention on both conditions in combination. Although prior studies have shown that up to half of near-centenarians and centenarians are at risk for social isolation [54], it is unclear whether or not they also suffer from loneliness, as findings are mixed. Although losses and life circumstances could make near-centenarians and centenarians more vulnerable [55], they have been found to be quite resilient and able to adjust their expectations to age-related challenges [56].

**Hypothesis** **1.** *Therefore, similar to younger old individuals [57], we expected that social isolation and loneliness would remain distinct phenomena with little to moderate overlap, meaning a near-centenarian or centenarian would be socially isolated without experiencing loneliness, or vice-versa.*

**Figure 1:**
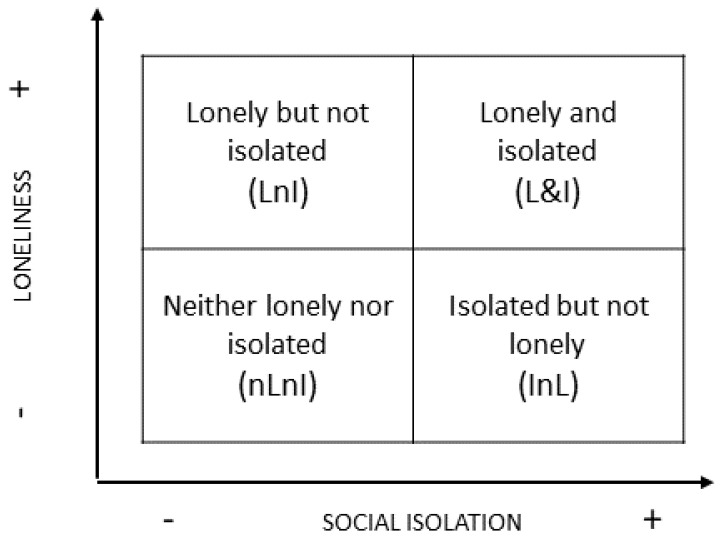
Combination of Social Isolation and Loneliness: Four-Group Typology.

Furthermore, drawing on Menec and colleagues [48] and Steptoe and colleagues [7], we differentiated between individuals who were isolated, lonely, or both from those who were not and combined them to create a four-group typology (see Figure 1).

**Hypothesis** **2a.** *Although researchers have found that the most disadvantaged group (“isolated and lonely”) represented a small minority, this combination may be more prevalent in near-centenarians and centenarians as a result of multiple losses.*

**Hypothesis** **2b.** *In turn, we expected that those who were neither isolated nor lonely represented a smaller part of the sample than in previous findings on younger-old people.*

**Hypothesis** **2c.** *We also expected the prevalence of isolated but not lonely individuals to be in line with that of studies on younger-old individuals because researchers have found that near-centenarians and centenarians are quite resilient, despite their restricted social networks.*

**Hypothesis** **2d.** *Then, we expected a higher prevalence of “lonely in the crowd” individuals than in findings for the younger-old, due to the high risk in very old age, even for those with extensive social resources, of losing their most meaningful relationships.*

Finally, we had an exploratory objective aimed at investigating which personal and environmental characteristics were associated with the probability of belonging to each group. Even though the studies on the combination of social isolation and loneliness and their correlates are limited, those examining social isolation and loneliness separately have highlighted correlations with variables from various domains (e.g., demographic, social, and socioeconomic) [18,19,20]. Drawing on these domains, we explored correlates of social isolation and loneliness, expecting them to be associated differently with each group. For instance, we expected living in a nursing home to be associated with feeling lonely without being socially isolated; good health to be associated positively with groups that do not feel lonely and negatively with those with high loneliness, regardless of the presence of isolation; high levels of extroversion or openness to be associated with less social isolation; and high neuroticism and low agreeableness to be associated with high loneliness.

## 2. Materials and Methods

### 2.1. Sample

We used data from the Fordham Centenarian Study. The main aim of this population-based study was to investigate physical, cognitive, social, and mental function in individuals aged 95 and older and to determine predictors of mental health indicators [54]. Recruitment was based on New York City’s voter registry. Of the 320 eligible individuals, after conducting 103 face-to-face interviews, 95 were included in the study (seven individuals had substantial cognitive impairment not obvious during the first contact; one withdrew after family intervention). A further 23 participants were contacted via healthcare providers. Finally, one additional centenarian was recruited by word of mouth. The final sample included 119 individuals aged from 95 to 107. For the present work, we included a total of 94 individuals with full information on key variables (i.e., non-missing values for loneliness and isolation measures). This subsample is similar to the whole Fordham Centenarian Study sample. Excluded participants (M = 13.4; SD = 5.1) had lower levels of cognitive functioning than included ones (M = 16.9; SD = 3.3; U = 3.13; *p* < 0.05), while no differences were found with regards to other characteristics (e.g., socio-demographic, health, or psychological) (See Table S1 in Supplementary Materials).

Study procedures were approved by three institutional review boards (Fordham University, Jewish Home Lifecare, and the Hebrew Home for the Aged).

### 2.2. Measures

#### 2.2.1. Social Isolation

Social isolation was assessed with the 6-item Lubben Social Network Scale (LSNS-6; [58]). The LSNS-6 includes three items for family and three for friends. Items enquire on how many relatives/friends the respondent talks to once a month, feels at ease with to talk about private matters, and feels close enough to call on them for help. Possible responses for each of the six LSNS-6 questions range from 0 (none) to 5 (nine or more). The possible total score ranges from 0 to 30), with higher scores indicating larger social networks. A score below 12 is a validated indicator of social isolation [58], which means that, on average, fewer than two individuals are available for the aspects of social networks assessed. The LSNS-6 was designed with the specific purpose of assessing social isolation in older adults and has been tested and used with oldest-old and very old populations [59,60]. Reliability in the present sample was high (Cronbach’s α = 0.77).

#### 2.2.2. Loneliness

Loneliness was assessed using five items from the UCLA Loneliness Scale [61]. This 5-item version has been tested and used with near-centenarians and centenarians [55]. Participants were asked to rate how often the following occurred: feeling unhappy doing many things alone, feeling that you have nobody to talk to, feeling that nobody really understands you, finding yourself waiting for people to call or write, and feeling completely alone. The responses (1 = never, 2 = rarely, 3 = sometimes, 4 = often) were averaged to create the loneliness score (range = 1–4), where higher scores indicated higher levels of loneliness. Reliability in the present sample was high (Cronbach’s α = 0.83).

#### 2.2.3. Demographic Indicators

We considered gender (0 = male, 1 = female), ethnicity (0 = White, 1 = Black), and chronological age (in years).

#### 2.2.4. Socioeconomic Indicators

We recoded education as a dichotomous variable (0 = less than high school diploma, 1 = high school diploma or more). Financial equity was measured with two single items (i.e., having difficulties meeting one’s needs, and covering medication costs; for both, 0 = no, 1 = yes).

#### 2.2.5. Social Indicators

We defined widowhood as a three-level variable, considering marital status together with length of widowhood (0 = no; 1 = yes, for less than 10 years; 2 = yes, for at least 10 years). Additional indicators included having living children (0 = yes, 1 = no), having living grandchildren (0 = no, 1 = yes), number of children living close by (0 = none, 1= one, 2 = more than one), living situation (0 = at home alone, 1 = at home with others, 2 = nursing home). Furthermore, we included the number of visits received per week (0 = no more than one visit, 1 = more than one). This indicator, unlike the LSNS-6, which measures significant and regular social contacts (e.g., help contacts, confidants, people seen regularly), instead collects information on all visits the participant may have had, even those on an infrequent or random basis (e.g., physician, cleaner, acquaintance). Finally, we considered satisfaction with how often family and friends come to visit (0 = no, 1 = yes). The latter two indicators had only moderate overlap and shared a limited amount of variance with social isolation (*χ*^2^ (1) = 11.47; *p* < 0.01; *Φ*^2^ = 0.125) and loneliness (*χ*^2^ (1) = 13.77; *p* < 0.01; *Φ*^2^ = 0.148), respectively. Therefore, we kept them in the model.

#### 2.2.6. Health Indicators

We coded the number of chronic diseases as a three-level variable (0 = 0–3, 1 = 4–5, 2 = 6 or more). Subjective health was measured with a single item asking participants to evaluate their current health status (1 = poor to 5 = excellent) and then recoded it into one of three categories (0 = poor–fair, 1 = good, 2 = very good–excellent). Functional health was evaluated using the seven instrumental activities of daily living (IADLs) from the Older Americans Resources and Services Multidimensional Functional Assessment Questionnaire [62]. Scores ranged from 0–14 with higher scores indicating better functional health. Because our sample had high values in basic activities of daily living (M = 10.4, SD = 3.7, range: 0–14), consistent with previous studies on the oldest-old and centenarians [63], we considered only instrumental activities of daily living to be indicators of functional status. We also measured how often the centenarians’ activities were restricted due to health conditions (0 = never or seldom; 1 = sometimes; 2 = often or always), how strong their physical pain was (range: 1–10), as well as the extent of their fatigue (measured by the mean of respondent’s answers to a four-item scale asking how often they have a lot of energy, feel tired, feel that everything requires an effort, feel to not get going). Response options ranged from 1 (=rarely or none of the time) to 4 (=all the time)., Given that recoding continuous covariates into categorical ones eases the interpretation of the results of nonlinear models typically used in study designs in which the dependent variable is not continuous [64], we recoded health-related continuous variables into new variables, creating (if not otherwise indicated) categories from tertiles of the scores distribution, where the third tertile represents the highest expression of the variable

#### 2.2.7. Care Indicators

The Fordham Centenarian Study included the number of the centenarians’ caregivers, recoded here as a dichotomous variable (0 = none or 1, 1 = more than 1) and differentiated between receiving help from professionals (0 = no, 1 = yes) or from informal providers, such as family or friends (0 = no, 1 = yes).

#### 2.2.8. Psychological Indicators

Life satisfaction was assessed with the 5-item Satisfaction with Life Scale [65], which uses 5 response options (from 0 = not at all to 4 = very much). Items were combined into a mean score, with higher values representing greater life satisfaction. Depressive symptomatology was measured with the 15-item version of the Geriatric Depression Scale [66], with response options including 1 = yes and 0 = no, added together, with higher values indicating more depressive symptoms. Personality traits were measured using a 10-item short version of the Big Five Inventory [67] with 2 items targeting each personality dimension. Possible responses ranged from 1 (strongly disagree) to 5 (strongly agree). Drawing on work of Ormstad and colleagues [68], who found significant differences in the association between loneliness and the lowest and highest values of the distribution of psychological indicators, we recoded all variables in this domain by calculating tertiles of the distribution. All the psychological measures used in the Fordham Centenarian Study showed good internal validity, as reported in previous publications [2,54].

### 2.3. Statistical Analysis

We analyzed descriptive data by using percentages and frequencies for categorical variables or mean and standard deviation for continuous variables. To address Hypothesis 1, we examined the relationship between social isolation and loneliness. As the Shapiro-Wilk test for measures of isolation (W = 0.96; *p* = 0.006) and loneliness (W = 0.94; *p* < 0.001) showed a significant departure from normality, we performed a Spearman correlation test. To verify Hypotheses 2a–2d, we combined the loneliness and isolation variables in a four-group typology. We calculated a dichotomous variable for loneliness, where 0 meant having a mean loneliness value equal to or below 2 (low loneliness), and 1 (mean value >2) meant high loneliness. To differentiate between individuals at risk and not at risk of social isolation, we used the aforementioned validated threshold of the LSNS-6.

We addressed our third exploratory aim using regression analysis. As the dependent variable had categories without natural ordering and the small sample size did not allow us to include all variables simultaneously in the models, we conducted several domain-specific multinomial logistic regression models. We included all measures belonging to a given domain as covariates in each domain-specific model (e.g., only health or psychological variables). Given the exploratory nature of this research goal, the model-building process was based mainly on a “background knowledge” approach [69] rather than on the “events-per-variable” criterion, which has only weak evidence supporting it [70]. Because age, gender, and ethnicity can affect several indicators across domains (e.g., education, widowhood, health, personality), we included them as control variables in each model, for which we set the confidence interval at 90% due to the small sample size. Furthermore, we checked for multicollinearity among covariates of each model by testing for variance inflation factor (VIF) and tolerance criterion, finding no problems of collinearity (see Table S2 in Supplementary Materials). As the parameters of a multiple-outcome model can be hard to interpret, we considered the results average marginal effects, (i.e., the average difference in probabilities across categories of a variable) [71]. We conducted all analyses using Stata 16.0, StataCorp (College Station, TX, USA) [72].

## 3. Results

Table 1 summarizes sample characteristics. Looking at the key measures of the study, the mean score of the LSNS-6 was 12.1 (SD = 0.40, range = 6–29), i.e., just above the validated threshold for isolation. The mean score for loneliness was 2.1 (SD = 0.90), which shows that participants rarely had feelings of loneliness, on average. We found a negative weak to moderate relationship between the UCLA loneliness scale and LSNS-6 total score (r_s_ (92) = −0.32, *p* < 0.05).

Table 2 shows the prevalence of the groups of the social isolation/loneliness typology. The two largest groups were “Lonely and Isolated (L&I)” (29.8%) and “Neither Lonely nor Isolated (nLnI)” (28.7%). The “Isolated but not Lonely (InL)” group represented 21.3% of the sample, and the “Lonely but not Isolated (LnI)” group represented 20.2%.

### Differential Group Characteristics

Table 3 summarizes a set of domain-specific multinomial logistic regressions and reports those covariates for which we found at least one significant average marginal effect (see Table S3 in Supplementary Materials for complete models).

Considering sociodemographic and social covariates, we found no statistically significant differences by gender or ethnicity. However, individuals with a high school diploma were, on average, 18% more likely than less educated ones to be LnI and 31% less likely to be L&I. Having grandchildren was related to a 22% higher likelihood of being nLnI. Compared to having those with no children living close by, individuals with one child living close by were 26% more likely to be in the InL group, and those with at least two were 29% more likely to be in the L&I group. Having met more than one person during the past week was related to a 22% higher likelihood of being in the nLnI group and an 18% reduced likelihood of being in the L&I group. Finally, being satisfied with family and friends’ visits was related to a higher likelihood of being in the nLnI or InL groups by 19% and 18%, respectively, and a 26% reduced likelihood of being in the L&I group.

Concerning health and care covariates, centenarians who reported very good or excellent subjective health were 27% less likely to be in the LnI group than those with poor health were. Reporting good health was associated with a 26% higher likelihood of being part of the InL group. Better functional health was associated with up to a 38% higher likelihood of being in the nLnI group and up to a 34% reduced likelihood of being in the L&I group. Health restrictions were related to a 23% lower likelihood of being in the nLnI group. Individuals with the highest levels of fatigue were 38% more likely to be in the InL group and 28% less likely to be in the nLnI group. Centenarians who received informal help were 30% more likely to be in the nLnI group and 19% less likely to be in the InL group. Individuals with more than one caregiver were 25% less likely to be nLnI and 33% more likely to be L&I.

Regarding psychological covariates, centenarians with moderate depressive symptoms were 22% more likely to be in the InL group than those in the first tertile were. In terms of personality dimensions, higher values of neuroticism were related to up to a 40% higher likelihood of being L&I and up to a 42% reduced likelihood of being InL. Higher values of openness were associated with up to a 32% higher likelihood of being nLnI and up to a 20% reduced likelihood of being InL.

As for agreeableness, centenarians in the second tertile were 21% more likely to be InL than those in the first tertile.

## 4. Discussion

The aim of this study was to investigate the combination of social isolation and loneliness in near-centenarians and centenarians.

Social isolation and loneliness are only weakly related among very old individuals in our sample. Extending work on younger old individuals [17] and supporting our first hypothesis, we found correlations of low effect size among both constructs, indicating that social isolation and loneliness are not identical in very old age. In other words, for a substantial number of individuals the two aspects did not converge. Although some overlap occurred between social isolation and loneliness in very old individuals, the weakness of the link suggests that the concepts do not converge more strongly in extreme old age than they do at younger ages (e.g., r = 0.201 in Coyle and Dugan [17]), and that even for individuals in very old age, we must consider these distinct concepts, which are not necessarily associated.

When we consider levels of social isolation and loneliness separately, our findings are consistent with those regarding the oldest-old [53,73]. In the present sample, near-centenarians and centenarians tended, on average, to have a number of meaningful social relationships just above the risk threshold and to report loneliness rarely. However, we found a different prevalence of the combination of social isolation and loneliness than has been found in studies on younger-old people [46,47,48]. Regarding Hypothesis 2a, our findings confirmed that the L&I group was, with almost 30% of the sample, the largest group. In studies with younger-old individuals, the prevalence was always below 10%, with the largest group constituted by those neither isolated nor lonely, representing at least half of those samples. Confirming Hypothesis 2b, the proportion of nLnI individuals was less than one third. The prevalence of InL individuals was in line with previous findings about younger-old individuals [46], confirming Hypothesis 2c. That very old individuals are more likely to be isolated is in line with prior studies’ conclusions that risk of social isolation increases for the oldest-old [4]. Regarding “lonely in the crowd” individuals, our findings confirm Hypothesis 2d: their prevalence was much higher among near-centenarians and centenarians than among younger-old individuals [48]. Despite some scholars considering loneliness a minority experience at very old age [27], our findings could indicate an increase in loneliness for the oldest-old, which is consistent with reports from centenarians indicating that the “worst side effect” of exceptional longevity is losing many loved ones, almost all friends and many family members, and that those losses are difficult to replace [55].

As expected, in our exploration of the association between groups and personal and environmental covariates we found specific characteristics associated with each group. The nLnI group was characterized by more social and care resources, better health, and an open-minded personality, which may favor establishing new relationships even in old age. This parallels prior studies [48]. Consistent with Smith and Victor’s findings [47], this group may be the least at risk of suffering adverse outcomes of social isolation and loneliness (i.e., low quality of life and mortality).

The L&I group was characterized by less education, poor functional health, and the presence of more than one caregiver. Although they had more children living close by, they were less satisfied with the frequency of their family and friends’ visits. This result may indicate that they failed to benefit from available social partners. This interpretation is in line with the higher levels of neuroticism of this group, as individuals with high neuroticism tend to feel more lonely [68] and less attached to those closest to them [25]. The higher prevalence of L&I individuals in very old age, in comparison to the prevalence studies have found in younger-old people [46,47,48], could result from a more precarious health status preventing more active engagement in social exchanges, the higher risk of losing loved ones at very old age, and the different predispositions to report negative phenomena across ages. Considering that loneliness and isolation are commonly thought of as widespread old age issues, centenarians may have been only somewhat affected by the social stigma that leads to underreporting negative conditions and feelings [74]. Loneliness in these very old individuals should be taken seriously; it would be very helpful to further investigate the underlying reasons and consider targeted interventions, which could include increasing social contacts, and opportunities to engage or provide support in everyday life tasks.

Turning to the InL individuals, despite having very limited social contacts, it seems they were satisfied with those they had. Even though they were more likely to have some health problems, report slightly increased depressive symptoms, and did not receive informal care, they did not experience loneliness. Living with a small social network may be a choice. Considering this group’s positive association with agreeableness and its negative association with high levels of openness, centenarians in this group may be in harmony with their existing (albeit scarce) social contacts while possibly also having issues in terms of opening themselves up to new social relationships. Their restricted networks may also be a result of recent changes, such as moving to a new environment or the loss of people to whom they were close. Despite these changes, which also may have led to depressive symptoms, these isolated centenarians may not have wanted to find new relationships, instead trying to make the most of their remaining social contacts. Not only does replacing close relationships become increasingly difficult with age [48], but some centenarians also reported that they just cannot replace some relationships [55]. Whether being socially isolated is a life choice or a recent adjustment, we may consider this group to be composed of resilient individuals able to optimize limited social relationships and to adjust their expectations. The strong negative association with neuroticism seems to confirm this interpretation. Indeed, being low in neuroticism is associated with a particularly resilient personality and a superior capacity to deal with stressful events [75]. Interventions for this group should take into account that simply providing more opportunities for social interaction may not be beneficial given that these individuals may not see the need to gain more social partners. Alternatively, ensuring access to support services (e.g., access to health care) may mitigate the risk of deterioration brought to centenarians’ resilience by worsening health conditions or the death of loved ones.

Regarding the LnI group, our results suggest that being “lonely in the crowd” is associated with having more education. This finding parallels those of prior studies, which have shown that education reduces the risk of social isolation and seems to offer more opportunities to have a large network of relationships, even at advanced ages [19]. This group membership was also associated with poorer subjective health. Previous evidence has demonstrated that subjective poor health is usually associated with less desirable personality traits, such as high neuroticism [76,77]. This group, despite its considerable number of meaningful social contacts, probably experienced loneliness due to a lack or loss of the most important relationships, a mismatch between quantity and quality of social contacts, or high expectations for their relationships [52]. To cope with the negative effects of loneliness, “lonely in the crowd” people may benefit from interventions aimed at making existing relationships more useful or enjoyable rather than increasing social contacts per se.

### Limitations

To the best of our knowledge, our study represents one of the first attempts to focus attention on the combination of social isolation and loneliness among the very old; however, there are some limitations that need consideration. A first limitation concerns the generalizability of our results. The small sample size could have affected the statistical significance of the results due to the low power, hiding associations between the four groups and individual characteristics in which we were interested. Therefore, taking into account the exploratory nature of our third goal, we set the confidence intervals at 90%, instead of the traditional 95% threshold. Furthermore, our findings may be restricted to near-centenarians and centenarians living in New York City: given geographical and cultural variations in the experience of social isolation and loneliness that. for instance, differentiate the spread and perceptions of social isolation and loneliness between collectivistic and individualistic societies [74,78,79], it is important to replicate our findings in other cultural settings. Despite the small sample size of the participant subgroup used for the present analyses, the Fordham Centenarian Study nonetheless represents a valuable source of information on the lives of the very old, who are often excluded or under-represented in larger population surveys. It also adopted a multidisciplinary approach using several internationally validated measures, such as those for social isolation and loneliness, often otherwise investigated with single questions.

A second limitation is due to the cross-sectional nature of the data, which does not allow us to make causal inferences. This means that we cannot assess whether the individual characteristics we have considered in fact predict or result from belonging to each of the four groups. Furthermore, we cannot verify whether social isolation or loneliness stem from conscious decisions (e.g., to restrict interactions or live isolated) or from critical life events (e.g., migration), since we have little information about such underlying aspects. Taking these issues into account, we adopted an exploratory approach aimed at evaluating the associations between social isolation and/or loneliness and individual characteristics.

Finally, the study sample may be affected by a selection effect. Oldest-old, and centenarians even more so, typically represent a hard to reach population, so their recruitment is challenging [80]. If non-participation was mainly linked to those conditions (e.g., poor health) usually associated with the highest values of social isolation or loneliness, then there may be an underestimation of the two phenomena. Furthermore, the exclusion of some participants due to missing values in key variables of social isolation and loneliness may bias our results. However, we checked for differences between included and excluded participants and found no statistically significant differences in the levels of almost all the individual characteristics, with the only exception being cognitive functioning.

## 5. Conclusions

Public and scientific debate on aging populations places increasing attention on social isolation and loneliness as key public health issues, especially with regard to the oldest-old. Combining social isolation and loneliness allows researchers to identify specific types of individuals who would otherwise remain hidden when both phenomena are investigated separately. With this exploratory study, we contribute to the existing literature by pointing out that near-centenarians and centenarians may be more frequently affected by social isolation and/or loneliness than younger-older adults, although mean levels of loneliness present in our sample could lead one to think that both—being isolated and feeling lonely—may not be widespread issues. Furthermore, we describe the combinations of social isolation and loneliness and their various associated individual characteristics. Identifying specific groups’ key characteristics can help design and improve person-centered interventions that address the unique needs of individuals belonging to each group.

The importance of distinguishing social isolation from loneliness and of distinguishing these phenomena from their combination should not be underestimated and deserves to be further investigated, ideally with qualitative methods able to grasp the meaning individuals attribute to particular life events (e.g., institutionalization or loss of affection) and to provide insight into how older people perceive their conditions. Researchers, therefore, need to investigate the combination of social isolation and loneliness in the very old, ideally by using longitudinal data and mixed methods to examine these aspects’ evolution over time. Furthermore, a multidisciplinary approach may be particularly useful to help increase the understanding of centenarians’ needs and provide well-tailored interventions reducing potential risk factors.

## Figures and Tables

**Table 1:** Characteristics of Study Participants.

Variables	*n*	*Mean (SD) or %*	*Min*	*Max*
*Key study variables*				
Social network (Lubben)				
Total score	94	12.2 (6.4)	3	29
Family score	94	7.1 (3.4)	0	15
Friends score	94	5.1 (4.4)	0	15
Loneliness (UCLA)	94	2.1 (0.9)	1	4
*Demographic variables*				
Gender				
Male	21	22.3		
Female	73	77.7		
Ethnicity				
White	75	80.6		
Black	18	19.4		
Age	94	99.6 (2.4)	95	107
*Socioeconomic variables*				
Education				
Less than diploma	48	51.6		
Diploma or more	45	48.4		
Difficulties living on income				
No	52	59.8		
Yes	35	40.2		
Difficulties paying medications				
No	69	76.7		
Yes	21	23.3		
*Social variables*				
Widowhood				
No	20	23.5		
Yes, for less than 10 years	13	15.3		
Yes, for at least 10 years	52	61.2		
Living children				
No	16	17.0		
Yes	78	83.0		
Having grandchildren				
No	31	33.0		
Yes	63	67.0		
Children living close				
No	46	48.9		
One	26	27.7		
More than one	22	23.4		
Living condition				
At home alone	49	52.1		
At home with others	25	26.6		
Nursing homes	20	21.3		
Meetings previous week				
No more than 1	38	41.3		
More than 1	54	58.7		
Satisfaction with family and friends meetings				
No	55	59.1		
Yes	38	40.9		
*Health variables*				
Chronic diseases				
0–3	25	26.6		
4–5	36	38.3		
6 or more	33	35.1		
Subjective health				
Poor/fair	29	30.8		
Good	34	36.2		
Very good/Excellent	31	33.0		
IADLs score	87	8.9 (4.0)	0	14
Restrictions due to health				
Never/Seldom	32	34.4		
Sometimes	24	25.8		
Often/Always	37	39.8		
Pain strength scale	92	4.4 (2.9)	1	10
Fatigue scale	93	2.2 (0.7)	1	4
*Care resources variables*				
Number of caregivers				
None or 1	53	56.4		
More than 1	41	43.6		
Professional help:				
No	22	23.4		
Yes	72	76.6		
Informal help				
No	53	56.4		
Yes	41	43.6		
*Psychological variables*				
Life satisfaction score	92	2.1 (1.2)	0	4
GDS	93	4.0 (3.5)	0	14
Personality traits				
Extraversion	93	3.1 (0.9)	1	5
Agreeableness	93	3.8 (0.9)	1	5
Conscienttiouness	93	3.9 (1.2)	1	5
Openness	93	3.8 (0.8)	2	5
Neuroticism	93	2.7 (1.2)	1	5

Notes: *n* = 94. *SD* = Standard Deviation. *Min* = Minimum value; *Max* = Maximum value.

**Table 2:** Social Isolation and Loneliness Groups.

Groups	*n*	*%*
Neither Lonely nor Isolated (nLnI)	27	28.7
Lonely but not Isolated (LnI)	19	20.2
Isolated but not Lonely (InL)	20	21.3
Lonely and Isolated (L&I)	28	29.8
Total	94	100.0

**Table 3:** Domain-specific Multinomial Logistic Regression Models: Significant Average Marginal Effects with 90% Confidence Intervals.

	Neither Lonely nor Isolated (nLnI)			Lonly but not Isolated (LnI)			Isolated but not Lonely (InL)			Lonely & Isolated (L&I)		
Models and Specific Covariates	AMEs	90% CI		AMEs	90% CI		AMEs	90% CI		AMEs	90% CI	
		LL	UL		LL	UL		LL	UL		LL	UL
Socioeconomic model												
Education ^a^												
Highschool diploma or more	-	-	-	0.175	0.035	0.315	-	-	-	−0.311	−0.468	−0.155
Social model												
Children living close ^b^												
One child	-	-	-	-	-	-	0.264	0.039	0.480	-	-	-
More than one	-	-	-	-	-	-	-	-	-	0.288	0.052	0.525
Meetings previous week ^c^												
More than one	0.246	0.071	0.422	-	-	-	-	-	-	−0.212	−0.399	−0.025
Satisfaction with family and friends’ meetings ^d^												
Yes	0.191	0.044	0.337	-	-	-	0.181	0.042	0.321	−0.260	−0.422	−0.099
Health model												
Subjective health ^e^												
Good	-	-	-	-	-	-	0.259	0.056	0.462	-	-	-
Very good/Excellent	-	-	-	−0.269	−0.478	−0.059	-	-	-	-	-	-
IADLs score ^f^												
2nd tertile	0.285	0.109	0.460	-	-	-	-	-	-	−0.224	−0.439	−0.009
3rd tertile	0.383	0.179	0.588	-	-	-	-	-	-	−0.340	−0.547	−0.132
Fatigue ^f^												
2nd tertile	-	-	-	-	-	-	-	-	-	-	-	-
3rd tertile	−0.276	−0.494	−0.059	-	-	-	0.381	0.145	0.616	-	-	-
Care resources model												
Number of caregivers ^g^												
More than 1	−0.246	−0.480	−0.013	-	-	-	-	-	-	0.330	0.159	0.502
Informal help ^h^												
Yes	0.295	0.069	0.522	-	-	-	−0.191	−0.374	−0.008	-	-	-
Psychological model												
Geriatric Depression Scale ^f^												
2nd tertile	-	-	-	-	-	-	0.225	0.043	0.406	-	-	-
3rd tertile	-	-	-	-	-	-	-	-	-	-	-	-
Agreeableness ^f^												
2nd tertile	-	-	-	-	-	-	0.209	0.047	0.370	−0.182	−0.354	−0.011
3rd tertile	-	-	-	-	-	-	-	-	-	-	-	-
Openness ^f^												
2nd tertile	0.355	0.194	0.516	-	-	-	-	-	-	-	-	-
3rd tertile	0.325	0.117	0.533	-	-	-	−0.202	−0.390	−0.014	-	-	-
Neuroticism ^f^												
2nd tertile	-	-	-	-	-	-	−0.242	−0.409	−0.074	0.212	0.072	0.351
3rd tertile	-	-	-	-	-	-	−0.418	−0.560	−0.275	0.407	0.233	0.581

Notes. CI = Confidence Interval; LL = Lower limit; UL = Upper limit. All models are controlled for age, gender, and ethnicity. AMEs = Average Marginal Effects. “-” = AMEs not statistically significant at 10% level (*p* ≥ 0.10). ^a^ 0 = Less than high school diploma. ^b^ 0 = none ^c^ 0 = no more than one. ^d^ 0 = no. ^e^ 0 = poor. ^f^ 0 = 1st tertile. ^g^ 0 = none or 1. ^h^ 0 = no.

## Data Availability

The data presented in this study are available on request from the authors.

## Supplementary Materials

The following supporting information can be downloaded at: https://www.mdpi.com/article/10.3390/ijerph19105940/s1, Table S1: Comparison between included and excluded participants by individual characteristics; Table S2: Variance inflations factors and tolerance criterion by domains; Table S3: Domain-specific multinomial logistic regression models: Average marginal effects with 90% confidence intervals.
