# Supplementary material for: Social Isolation and Loneliness among Near-Centenarians and Centenarians: Results from the Fordham Centenarian Study
Source: Int J Environ Res Public Health. 2022 May 13;19(10):5940. doi: 10.3390/ijerph19105940 (PMC9141482; doi:10.3390/ijerph19105940)
Supplement: Supplementary file 1 [file ijerph-19-05940-s001.zip › ijerph-1633097-supplementary.pdf]

Table S1. Comparison between included and excluded participants by individual characteristics

	Included		Excluded		
**Variables**	*n*	*Mean (SD) or %*	*n*	*Mean (SD) or %*	*p*
Demographic variables					
Gender					
Male	21	22.3	5	20.0	0.801
Female	73	77.7	20	80.0	
Ethnicity					
White	75	80.6	20	80.0	0.942
Black	18	19.4	5	20.0	
Age	94	99.6 (2.4)	25	99.3 (2.7)	0.913
Socio-economic variables					
Education					
Less than diploma	48	51.6	12	48.0	0.785
Diploma or more	45	48.4	13	52.0	
Difficulties living on income					
No	52	59.8	11	57.9	0.880
Yes	35	40.2	8	42.1	
Difficulties paying medications					
No	69	76.7	14	73.7	0.782
Yes	21	23.3	5	26.3	
Social variables					
Marital status					
Never married	9	9.6	2	8.0	0.291
Married	5	5.3	3	12.0	
Divorced	6	6.4	3	12.0	
Separate	1	1.1	1	4.0	
Widowed	73	77.7	16	64.0	
Living condition					
At home alone	49	52.1	15	60.0	0.625
At home with others	25	26.6	7	28.0	
Nursing homes	20	21.3	3	12.0	
Health variables					
Number of chronic diseases	94	4.9 (2.3)	22	4.8 (2.5)	0.857
IADLs score	87	8.9 (4.0)	15	8.8 (1.1)	0.924
Restrictions due to health					
Never/Seldom	32	34.4	9	37.5	0.878
Sometimes	24	25.8	5	20.8	
Often/Always	37	39.8	10	41.7	
Mini-mental state examination	94	16.9 (3.3)	25	13.4 (5.2)	0.006
Psychological variables					
Life satisfaction score	92	2.1 (1.2)	20	1.8 (1.2)	0.269
Geriatric Depression Scale	93	4.0 (3.5)	14	4.6 (3.1)	0.350

Table S2. Variance Inflations Factors (VIF) and Tolerance Criterion by domains

	VIF	Tolerance
Demographics domain		
Age	1.02	0.976
Gender	1.02	0.977
Ethnicity	1.01	0.995
Socio-economics domain		
Education	1.03	0.966
Difficulties with income	1.35	0.742
Difficulties paying meds meds	1.07	0.728
Social domain		
Widowhood	1.36	0.734
Living children	1.07	0.933
Having grandchildren	1.52	0.656
Children living close	1.50	0.665
Living condition	1.12	0.891
Meetings previous week	1.33	0.754
Satisfaction with family and friends meetings	1.36	0.734
Health domain		
Chronic diseases	1.20	0.831
Subjective health	1.25	0.801
IADLs score	1.21	0.824
Restrictions due to health	1.57	0.638
Pain	1.58	0.639
Fatigue	1.23	0.814
Care resources domain		
Number of caregivers	1.63	0.614
Professional help	1.79	0.559
Informal help	1.80	0.555
Psychological domain		
Life satisfaction	*1.26*	0.793
Geriatric Depression Scale	*1.33*	0.754
Extraversion	*1.33*	0.752
Agreeableness	1.07	0.937
Conscientiousness	1.09	0.915
Openness	1.07	0.935
Neuroticism	1.30	0.772

Table S3. Domain-specific Multinomial Logistic Regression Domains: Average Marginal Effects with 90% Confidence Intervals. *

Domains and specific correlates	Neither Lonely Nor Isolated			Lonly but not Isolated			Isolated but not Lonely			Lonely & Isolated		
	**AMEs**	**90% CI**		**AMEs**	**90% CI**		**AMEs**	**90% CI**		**AMEs**	**90% CI**	
		LL	UL		LL	UL		LL	UL		LL	UL
Demographics domain												
Age	−0.006	−0.039	0.027	−0.009	−0.038	0.020	−0.008	−0.037	0.021	0.023	−0.010	0.056
Gender												
Male (ref.)												
Female	−0.095	−0.279	0.088	−0.013	−0.178	0.152	−0.015	−0.185	0.156	0.123	−0.084	0.330
Ethnicity												
White (ref)												
Black	0.063	−0.142	0.269	−0.033	−0.199	0.133	0.093	−0.099	0.285	−0.124	−0.300	0.053
Socio-economics domain [a]												
Education												
Less than highschool diploma (ref.)												
Highschool diploma or more	0.123	−0.044	0.290	**0.175**	**0.035**	**0.315**	0.013	−0.136	0.163	**−0.311**	**−0.468**	**−0.155**
Difficulties with income												
No (ref.)												
Yes	−0.039	−0.229	0.150	0.004	−0.154	0.162	0.157	−0.017	0.332	−0.122	−0.299	0.055
Difficulties paying meds meds												
No (ref.)												
Yes	0.126	−0.126	0.377	−0.026	−0.218	0.165	−0.146	−0.306	0.014	0.046	−0.179	0.271
Social domain [a]												
Widowhood												
No (ref.)												
Yes, for less than 10 years	0.029	−0.245	0.303	0.207	−0.047	0.461	−0.211	−0.455	0.033	−0.025	−0.297	0.247
Yes, for at least 10 years	0.060	−0.160	0.279	0.059	−0.091	0.209	−0.101	−0.339	0.137	−0.017	−0.237	0.203
Living children												
Yes (ref.)												
No	−0.047	−0.288	0.193	0.030	−0.167	0.227	−0.113	−0.340	0.115	0.130	−0.082	0.341

Domains and specific correlates	Neither Lonely Nor Isolated AMEs	90% CI LL	90% CI UL	Lonly but not Isolated AMEs	90% CI LL	90% CI UL	Isolated but not Lonely AMEs	90% CI LL	90% CI UL	Lonely & Isolated AMEs	90% CI LL	90% CI UL
Having grandchildren												
No (ref.)												
Yes	0.240	0.058	0.423	0.031	−0.118	0.180	−0.141	−0.332	0.050	−0.130	−0.337	0.076
Children living close												
None (ref.)												
One	−0.180	−0.379	0.019	−0.069	−0.263	0.126	**0.264**	**0.039**	**0.480**	−0.015	−0.217	0.186
More than one	−0.186	−0.377	0.006	−0.154	−0.328	0.020	0.051	−0.132	0.233	**0.288**	**0.052**	**0.525**
Living condition												
At home alone (ref.)												
At home with others	0.069	−0.130	0.268	−0.017	−0.193	0.159	−0.151	−0.304	0.003	0.098	−0.088	0.284
Nursing home	−0.120	−0.317	0.078	−0.024	−0.190	0.142	−0.005	−0.224	0.214	0.148	−0.059	0.356
Meetings previous week												
No more than one (ref.)												
More than one	**0.246**	**0.071**	**0.422**	0.109	−0.048	0.266	−0.144	−0.313	0.026	**−0.212**	**−0.399**	**−0.025**
Satisfaction with family and friends meetings												
No (ref.)												
Yes	**0.191**	**0.044**	**0.337**	−0.112	−0.260	0.036	**0.181**	**0.042**	**0.321**	**−0.260**	**−0.422**	**−0.099**
Health domain [a]												
Chronic diseases												
0 to 3 (ref.)												
4 or 5	0.039	−0.149	0.228	0.052	−0.137	0.241	0.067	−0.122	0.257	−0.158	−0.346	0.029
6 or more	0.048	−0.158	0.254	−0.055	−0.234	0.124	−0.009	−0.204	0.185	0.016	−0.202	0.233
Subjective health												
Poor (ref.)												
Good	−0.033	−0.236	0.171	−0.205	−0.427	0.017	**0.259**	**0.056**	**0.462**	−0.021	−0.200	0.157
Very good/Excellent	0.162	−0.069	0.392	**−0.269**	**−0.478**	**−0.059**	0.022	−0.130	0.174	0.085	−0.098	0.268
IADLs score												
1st tertile (ref.)												
2nd tertile	**0.285**	**0.109**	**0.460**	0.031	−0.152	0.213	−0.091	−0.275	0.092	**−0.224**	**−0.439**	**−0.009**

Domains and specific correlates	Neither Lonely Nor Isolated AMEs	90% CI LL	90% CI UL	Lonly but not Isolated AMEs	90% CI LL	90% CI UL	Isolated but not Lonely AMEs	90% CI LL	90% CI UL	Lonely & Isolated AMEs	90% CI LL	90% CI UL
3rd tertile	**0.383**	**0.179**	**0.588**	−0.059	−0.253	0.136	0.015	−0.205	0.235	**−0.340**	**−0.547**	**−0.132**
Restrictions due to health												
Never, seldom (ref.)												
Sometimes	0.078	−0.165	0.320	−0.082	−0.275	0.110	0.031	−0.168	0.230	−0.026	−0.220	0.167
Often, always	−0.229	−0.422	−0.035	0.000	−0.204	0.205	0.156	−0.039	0.351	0.072	−0.119	0.264
Pain												
1st tertile (ref.)												
2nd tertile	0.132	−0.068	0.331	−0.048	−0.246	0.150	−0.014	−0.231	0.203	−0.070	−0.255	0.116
3rd tertile	0.091	−0.105	0.288	−0.153	−0.328	0.022	−0.124	−0.314	0.065	0.186	−0.038	0.411
Fatigue												
1st tertile (ref.)												
2nd tertile	−0.174	−0.378	0.030	0.154	−0.037	0.346	0.055	−0.093	0.203	−0.035	−0.214	0.143
3rd tertile	**−0.276**	**−0.494**	**−0.059**	−0.110	−0.263	0.044	**0.381**	**0.145**	**0.616**	0.005	−0.234	0.245
Care resources domain [a]												
Number of caregivers												
None or 1 (ref.)												
More than 1	**−0.246**	**−0.480**	**−0.013**	−0.041	−0.230	0.147	−0.043	−0.215	0.130	**0.330**	**0.159**	**0.502**
Professional help												
No (ref.)												
Yes	0.089	−0.158	0.335	0.101	−0.125	0.326	−0.138	−0.327	0.051	−0.051	−0.281	0.179
Informal help												
No (ref.)												
Yes	**0.295**	**0.069**	**0.522**	0.055	−0.137	0.246	−0.191	−0.374	−0.008	**−0.159**	**−0.344**	**0.026**
Psychological domain [a]												
Life satisfaction												
1st tertile (ref.)												
2nd tertile	0.083	−0.103	0.269	−0.068	−0.249	0.114	−0.068	−0.248	0.112	0.053	−0.107	0.212
3rd tertile	0.068	−0.124	0.259	−0.031	−0.235	0.173	−0.018	−0.221	0.185	−0.019	−0.216	0.178
Geriatric Depression Scale												

Domains and specific correlates	Neither Lonely Nor Isolated			Lonly but not Isolated			Isolated but not Lonely			Lonely & Isolated		
	AMEs	90% CI		AMEs	90% CI		AMEs	90% CI		AMEs	90% CI	
		LL	UL		LL	UL		LL	UL		LL	UL
1st tertile (ref.)												
2nd tertile	−0.058	−0.259	0.143	−0.031	−0.227	0.165	**0.225**	**0.043**	**0.406**	−0.136	−0.296	0.024
3rd tertile	−0.182	−0.387	0.024	−0.159	−0.340	0.022	0.154	−0.045	0.354	0.186	−0.029	0.402
Extraversion												
1st tertile (ref.)												
2nd tertile	0.031	−0.158	0.220	−0.094	−0.292	0.104	−0.067	−0.220	0.086	0.129	−0.023	0.282
3rd tertile	0.043	−0.150	0.236	−0.176	−0.375	0.024	0.041	−0.148	0.231	0.091	−0.085	0.267
Agreeableness												
1st tertile (ref.)												
2nd tertile	−0.117	−0.294	0.060	0.091	−0.101	0.282	**0.209**	**0.047**	**0.370**	**−0.182**	**−0.354**	**−0.011**
3rd tertile	0.120	−0.068	0.308	−0.072	−0.230	0.085	0.029	−0.131	0.190	−0.078	−0.261	0.105
Conscientiousness												
1st tertile (ref.)												
2nd tertile	0.025	−0.211	0.260	0.125	−0.049	0.298	0.020	−0.185	0.226	−0.170	−0.371	0.031
3rd tertile	0.097	−0.143	0.336	0.117	−0.053	0.287	−0.073	−0.246	0.099	−0.140	−0.348	0.067
Openness												
1st tertile (ref.)												
2nd tertile	**0.355**	**0.194**	**0.516**	−0.153	−0.344	0.038	0.006	−0.186	0.199	−0.208	−0.418	0.003
3rd tertile	**0.325**	**0.117**	**0.533**	0.092	−0.188	0.371	**−0.202**	**−0.390**	**−0.014**	−0.215	−0.460	0.029
Neuroticism												
1st tertile (ref.)												
2nd tertile	−0.012	−0.179	0.154	0.042	−0.124	0.207	**−0.242**	**−0.409**	**−0.074**	**0.212**	**0.072**	**0.351**
3rd tertile	0.006	−0.186	0.199	0.004	−0.170	0.179	**−0.418**	**−0.560**	**−0.275**	**0.407**	**0.233**	**0.581**

Notes. CI = Confidence Interval; .LL= Lower limit; UL= Upper limit. AMEs = Average Marginal Effects. (ref.) = reference category.

[*] Bold values =AMEs significant at 10% level ($p < 0.10$).

[a] Domain controlled for age, gender, and ethnicity.
